# Remodeling of the neutrophil proteome upon exposure to *Pseudomonas aeruginosa* biofilms

**DOI:** 10.1128/mra.01147-24

**Published:** 2025-03-13

**Authors:** Tia Rizakos, Jennifer Geddes-McAlister

**Affiliations:** 1 Molecular and Cellular Biology Department, University Of Guelph 317113 , Guelph, Ontario, Canada; University of Notre Dame, Notre Dame, Indiana, USA

**Keywords:** proteomics, neutrophils, *Pseudomonas aeruginosa*, biofilms

## Abstract

Neutrophils play important roles within the innate immune system as the first line of defense against invading pathogens. Pathogens counteract these defenses through multiple mechanisms, including protective biofilm formation. Herein, we assessed proteome remodeling between *Pseudomonas aeruginosa* biofilms and HL-60 cells to provide new insights into host defense responses.

## ANNOUNCEMENT

Neutrophils account for 70% of circulating leukocytes and act as the first line of defense against invading pathogens by engaging in phagocytosis, degranulation, and neutrophil extracellular trap formation (1–3). *Pseudomonas aeruginosa* is a Gram-negative bacterium that forms biofilms (i.e., high-density bacterial clusters attached to surfaces) to provide protection from the immune system, antibiotic treatment, and antimicrobials (4–6). Neutrophils are essential to clear *P. aeruginosa* biofilms and infections through the actions of antimicrobial proteins and peptides (6–11).

Herein, we evaluated proteome remodeling of the host following exposure of neutrophils (differentiated from HL-60 cells) to *P. aeruginosa* biofilms. HL-60 cells were cultured in suspension in Roswell Park Memorial Institute (RPMI) 1640 medium supplemented with 10% fetal bovine serum, 1% L-glutamine, and 1% antibiotic-antimycotic (Anti-Anti) (12). Cells were maintained at 37 °C and 5% CO_2_, passaged 5–15 times, and exposed to 1.25% dimethyl sulfoxide RPMI-supplemented media every 2 days to promote neutrophil differentiation (12). Neutrophils were collected by centrifugation at 400 × *g* for 4 min, washed twice in phosphate-buffered saline (pH 7.4), and resuspended in RPMI 1640 medium (without Anti-Anti). Laboratory-associated *P. aeruginosa* PAO1 strain (ATCC: BAA-47) was used for bacterial biofilms. A single PAO1 colony was used to inoculate 5 mL of Lysogeny broth (LB) at 37 °C with shaking (200 rpm) followed by optical density normalization to 0.5 and the addition of 5 µL of culture to 200 µL of LB grown statically for 24 h at 37 °C. Biofilm formation was confirmed by crystal violet assay (13). Neutrophils were placed on top of biofilms at a multiplicity of infection of 1:100 (neutrophils: PAO1 biofilms) for 4 h at 37 °C and 5% CO_2_.

*P. aeruginosa* biofilms and co-cultured neutrophils were collected by water bath sonication followed by cell scraping and centrifugation at 400 × *g* for 4 min. Separately, neutrophils not exposed to biofilms were also collected by centrifugation at 400 × *g* for 4 min as controls. Samples (i.e., neutrophils and bacterial biofilms or neutrophils only) were prepared in biological quadruplicate, and protein extraction was performed as we previously described with trypsin/LysC digestion of 25 µg (determined by tryptophan assay [14]) of protein (15). Peptides (3 µg) were subjected to nanoflow liquid chromatography on an Easy-nLC 1200 system (Thermo Fisher Scientific) coupled to Orbitrap Exploris 240 hybrid quadrupole-orbitrap mass spectrometer (Thermo Fisher Scientific). Peptide samples were loaded into a 50 cm PepMap RSLC EASY-Spray column (75 µM inner diameter) filled with 2 µm C18 reverse-phase silica beads (Thermo Fisher Scientific) and electrosprayed with a linear gradient of 3%–20% Buffer B over 2 h at 250 nL/min. The mass spectrometer was operated using data-dependent acquisition, and full scans (*m/z* 400 to 2,000) were acquired at 120,000 resolution. Raw data files were processed with MaxQuant (version 1.6.17) (16) with the Andromeda search engine against *Homo sapiens* (82,492 sequences; December 2022) and *P. aeruginosa* (strain ATCC 15692/PAO1) (5,564 sequences; December 2022) FASTA files. Label-free quantification ratio = 1, Match Between Runs was enabled, minimum number of peptides = 2, and split by taxonomy ID was enabled. All other parameters were as default (i.e., fixed modification of carbamidomethylation, variable modification of oxidation and acetylation). Data were analyzed and visualized using Perseus (version 1.6.2.2) (17), filtered to remove contaminants, reverse peptides, and peptides only identified by site and log_2_ transformed. Rows were filtered based on valid values (three of four replicates) and imputed from the normal distribution (width of 0.3 and a downshift of 1.8). Significant differences in protein abundance were determined by Student’s *t*-test (*P* value ≤0.05) with multiple hypothesis testing correction using the Benjamini-Hochberg false discovery rate (FDR) (FDR = 0.01) and S_0_ = 1 (18).

**Fig 1:**
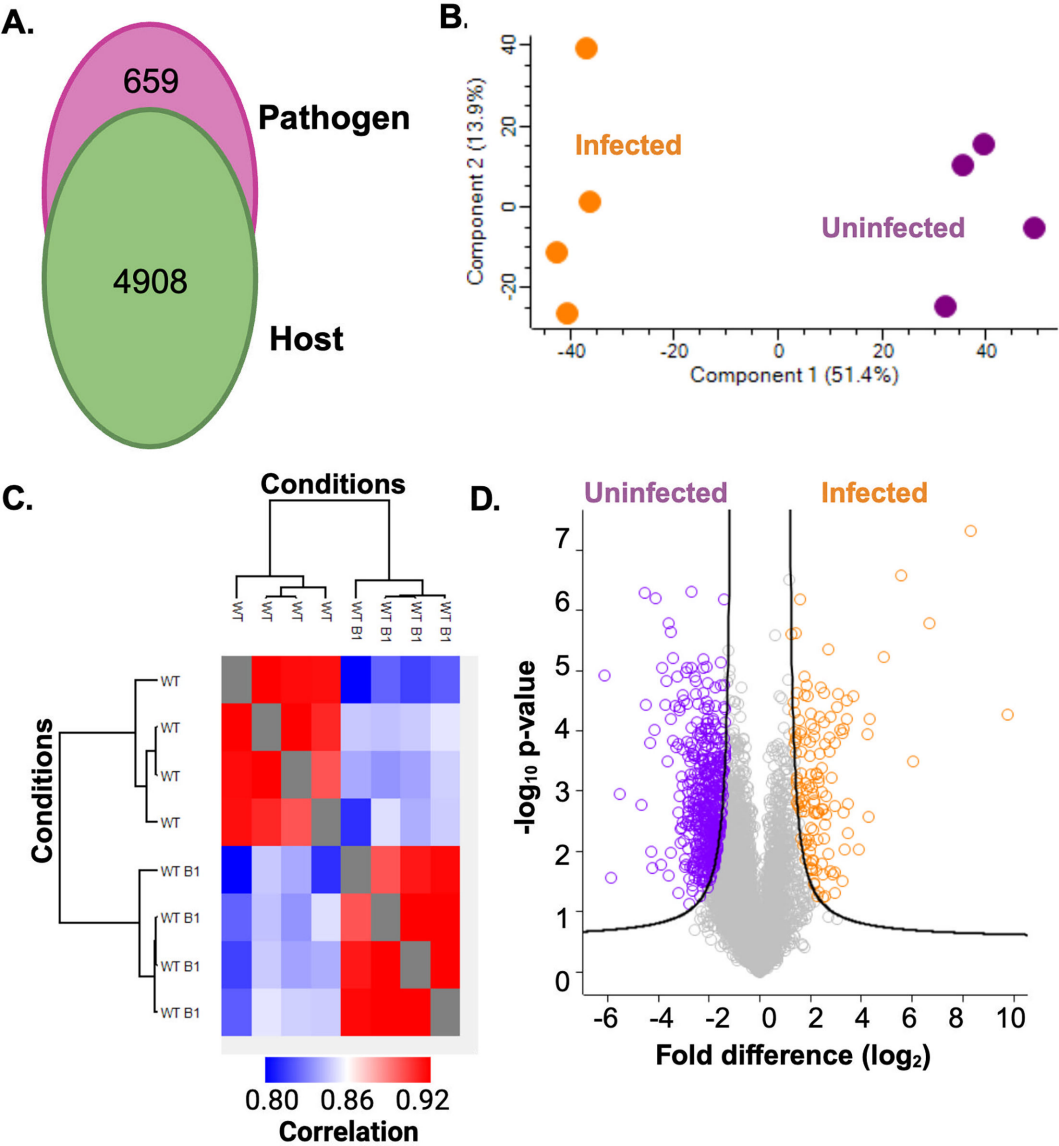
Proteome profiling of neutrophils co-cultured with *P. aeruginosa* biofilms. (A) Number of total proteins identified from each biological system. (B) Principal component analysis of host proteome. (C) Heat map of hierarchical clustering by Euclidean distance for host proteins. WT, uninfected; WT B, infected. (D) Volcano plot of host proteins. FDR-corrected Student’s *t*-test *P* value <0.05, FDR = 0.01, S_0_ = 1. Performed in biological quadruplicate.

We identified 4,908 host proteins and 659 bacterial proteins (Fig. 1A), and a principal component analysis of host proteins demonstrated clustering between infected and uninfected samples in the dimension of component 1 (51.4%) and separation of biological replicates along component 2 (13.9%) (Fig. 1B). A host protein heat map for hierarchical clustering by Euclidean distance showed replicate reproducibility at 91.5% (neutrophils only) and 92.1% (neutrophils co-cultured with *P. aeruginosa*) (Fig. 1C). A volcano plot defined 475 host proteins with a significant increase in abundance for uninfected neutrophils compared to 132 host proteins with a significant increase in abundance during infection, including key proteins associated with immune response (e.g., neutrophil elastase) (Fig. 1D) (19). Overall, this study provides new insights into proteome remodeling of neutrophils upon exposure to *P. aeruginosa* biofilms and supports further investigations into biological drivers of host defense.

## Data Availability

The mass spectrometry-based proteomics data is available through PRIDE Proteome Exchange with accession number PXD056790.
